# Comparative genomics reveals conservation of filaggrin and loss of caspase-14 in dolphins

**DOI:** 10.1111/exd.12681

**Published:** 2015-04-27

**Authors:** Bettina Strasser, Veronika Mlitz, Heinz Fischer, Erwin Tschachler, Leopold Eckhart

**Affiliations:** Research Division of Biology and Pathobiology of the Skin, Department of Dermatology, Medical University of ViennaVienna, Austria

**Keywords:** caspase-14, evolution, filaggrin, protease, skin barrier

## Abstract

The expression of filaggrin and its stepwise proteolytic degradation are critical events in the terminal differentiation of epidermal keratinocytes and in the formation of the skin barrier to the environment. Here, we investigated whether the evolutionary transition from a terrestrial to a fully aquatic lifestyle of cetaceans, that is dolphins and whales, has been associated with changes in genes encoding filaggrin and proteins involved in the processing of filaggrin. We used comparative genomics, PCRs and re-sequencing of gene segments to screen for the presence and integrity of genes coding for filaggrin and proteases implicated in the maturation of (pro)filaggrin. Filaggrin has been conserved in dolphins (bottlenose dolphin, orca and baiji) but has been lost in whales (sperm whale and minke whale). All other S100 fused-type genes have been lost in cetaceans. Among filaggrin-processing proteases, aspartic peptidase retroviral-like 1 (ASPRV1), also known as saspase, has been conserved, whereas caspase-14 has been lost in all cetaceans investigated. In conclusion, our results suggest that filaggrin is dispensable for the acquisition of fully aquatic lifestyles of whales, whereas it appears to confer an evolutionary advantage to dolphins. The discordant evolution of filaggrin, saspase and caspase-14 in cetaceans indicates that the biological roles of these proteins are not strictly interdependent.

## Introduction

The stratum corneum provides terrestrial vertebrates with an indispensable protection against the dry environment. Intracellular remodelling of epidermal keratinocytes, also known as cornification, proteinaceous connections between terminally differentiating cells, and the establishment of an extracellular lipid compartment are critical processes in establishment of the barrier against water loss and to the entry of noxious substances from the environment (1–3).

Genetic studies have suggested that one of the crucial proteins for the human skin barrier is filaggrin. Mutations in the filaggrin (*FLG*) gene are associated with atopic dermatitis and ichthyosis vulgaris (4–6). Knockout of *FLG* in the mouse and siRNA-mediated knockdown of *FLG* expression in human skin equivalents impair epidermal barrier functions (7,8). Filaggrin is an intracellular protein that aggregates intermediate filaments *in vitro* and *in vivo* (9–11), hence its name, which is short for filament aggregating protein. In addition, filaggrin has been suggested to contribute to the degradation of the nucleus during cornification of keratinocytes (11,12). Moreover, filaggrin is a major source of amino acids that constitute, either directly or after modification, the so-called natural moisturizing factor of the stratum corneum (13–15). In particular, the breakdown of filaggrin (also termed histidine-rich protein) releases histidine that is converted to urocanic acid, a natural sunscreen of the skin (16). Proteases such as saspase (17) and caspase-14 (18–21) are implicated in the stepwise proteolytic processing of filaggrin.

Filaggrin belongs to the family of S100 fused-type proteins (SFTPs), which are characterized by the presence of an amino-terminal S100 domain with calcium-binding activity, a long central domain containing sequence repeats and a carboxy-terminus that differs from central sequence repeats (22–24). Proteolytic processing of the full-length precursor protein (also referred to as profilaggrin) releases the so-called filaggrin monomers, which essentially correspond to the central domain sequence repeats (23,25). Other human SFTPs are filaggrin 2, hornerin, cornulin, repetin, trichohyalin and trichohyalin-like 1 (23,25). Recently, trichohyalin-like 2 has been reported for sheep and other mammals (26). The recent identification of SFTPs in sauropsids (reptiles and birds) (24) has suggested that SFTPs originated before the divergence of the evolutionary lineages leading to modern mammals and sauropsids approximately 310 million years ago. No SFTPs have been identified in fishes and amphibians, indicating the origin of SFTPs was associated with the evolutionary water-to-land transition of a subgroup of vertebrates known as amniotes. Filaggrin and caspase-14 are restricted to mammals (24,27), possibly indicating co-evolution of components of a filaggrin-dependent skin barrier system in mammals.

While it is evident that the epidermal barrier to water loss in the dry environment was a key innovation during the evolution of the terrestrial lifestyle of amniotes (28,29), the fate of the skin barrier during the return of some of the terrestrial vertebrates to a fully aquatic lifestyle is less clear (30). The mammalian clade of the cetaceans is comprised of dolphins and whales. In this manuscript, the term ‘dolphins’ refers to the phylogenetic clade comprising the oceanic dolphins (bottlenose dolphin, orca and others) and the river dolphins (including the Yangtze river dolphin, also known as baiji, and others), whereas ‘whales’ refers to the paraphyletic group within the order of cetaceans that is phylogenetically basal to the dolphins as defined above and includes the sperm whale, the minke whale and others (Fig. S1). Only few aspects of the skin biology of cetaceans have been addressed in previous studies, with particularly little information about molecular biology of cetacean skin being available. Cetaceans are exposed to the air only during short periods of time when they are breathing. Thus, the hydration of their outermost skin layers does not require hydration mechanisms active in the epidermis of terrestrial mammals. However, the hyperosmotic environment of marine mammals and the hypo-osmotic environment of river dolphins necessitate permeability barrier functions to control the water flux through the epidermis (31). The epidermis of cetaceans is several millimetres thick and rich in deep papillae indicative of high proliferative activity in an extended basal layer. A stratum corneum is not well defined in cetaceans, and the superficial cells contain nuclei (parakeratosis) (Fig. S2) (32,33). Keratohyalin granules are absent in cetacean epidermis (33). Instead of desquamation of individual corneocytes, large pieces of the outermost epidermal layers flake off from the surface of cetaceans.

Here, we have applied comparative genomics to investigate the possible adaptations of filaggrin and two filaggrin-processing proteases (caspase-14 and saspase) during or after the evolutionary transition of cetaceans from terrestrial to fully aquatic life. We show that filaggrin has been conserved in dolphins, but lost in whales and that caspase-14 and saspase have not co-evolved with filaggrin in cetaceans.

## Material and methods

### Comparative genomics

The genome sequences of bottlenose dolphin (*Tursiops truncatus*) (34), orca (*Orcinus orca*), Yangtze river dolphin (*Lipotes vexillifer*) (35), sperm whale (*Physeter catodon*), minke whale (*Balaenoptera acutorostrata scammoni*) (36), cattle (*Bos taurus*) and humans (*Homo sapiens*) were investigated for the presence and sequence integrity of genes encoding SFTPs, caspases and saspase. In addition, distinct regions of the genome sequences of other species were used for sequence comparisons. The sequences were retrieved from the GenBank database of the National Center for Biotechnology Information (NCBI), USA (http://www.ncbi.nlm.nih.gov/). Gene predictions and sequence alignments were performed essentially using an approach described previously (29,37). The Basic Local Alignment Search Tool (BLAST) was used to search for regions of local similarity between sequences. The conservation of blocks of order of genetic elements (synteny) was tested by manual alignment of gene maps, focusing on a region including between 2 and 5 genes on each side the gene(s) of interest. Nucleotide and amino acid sequences were aligned using the programs BLAST and Multalin (38).

### Tissue and DNA samples

Skin samples from stranded individuals of the bottlenose dolphin (*Tursiops truncatus*) (SW1999/197) and the harbour porpoise (*Phocoena phocoena*) (SW2002/382) were kindly provided by Rob Deaville, Zoological Society of London, London, UK. The samples were originally stored in ethanol and later processed for histology and DNA extraction according to published protocols (37). DNA from the fin whale (*Balaenoptera physalus*) and the hippopotamus (*Hippopotamus amphibius*) was kindly provided by Michael Wallis, Biochemistry Department, University of Sussex, Brighton, UK. Tissue samples from pig and cattle were kindly provided by Wolfgang Sipos, Clinical Department for Farm Animals and Herd Management, University of Veterinary Medicine, Vienna, Austria.

### PCR screening for the presence of the *CASP14* gene

The presence of a caspase-14 gene was tested by PCR using primers annealing to conserved sequence sites. The primer pairs were 5′-AGGTGACCCGGCGGATGGC-3′ and 5′-TACTGCAGATA*N*AG*NY*GTTTCCG-3′ for the PCR ‘CASP14-1′ and 5′-TA*Y*GACATGTC*N*GGTGCCCGCCT-3′ and 5′-TTCAT*S*GTGCTCTCAAA*N*C*Y*CAGCTG-3′ for the PCR ‘CASP14-2′ where the degenerate base symbols are *Y* for pyrimidine (C or T), *S* for strong binders (C or G) and *N* for any base. To confirm the integrity of the genomic DNAs, an evolutionarily conserved segment of the prion protein (*PRNP*) gene was amplified using primers reported previously (39).

## Results

### Filaggrin genes are conserved in dolphins but not in whales

We screened the draft genome sequences of cetaceans as well as those of their closest terrestrial relative with a sequenced genome, that is the cattle (Table S1), for genes encoding S100 fused-type proteins (filaggrin and others), caspases and saspase. Gene predictions were based on BLAST similarity searches and comparisons of syntenic loci in terrestrial mammals and cetacean. The validity of assembled genome sequence of the bottlenose dolphin was tested by resequencing several regions of interest.

**Figure 1:**
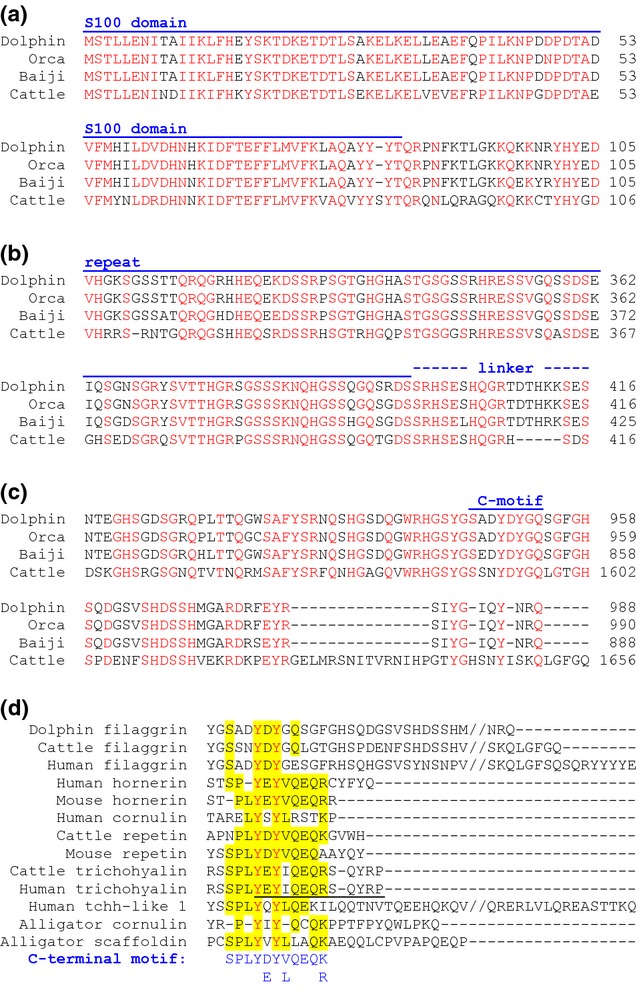
Dolphins have filaggrin proteins with conserved amino acid sequence elements. Amino acid sequences of filaggrin proteins of dolphin (*Tursiops truncatus*), orca (*Orcinus orca*), baiji (*Lipotes vexillifer*) and cattle (*Bos taurus*) were aligned. The sequence alignments corresponding to the N-terminus (a), representative repeat and linker segments of the central region (b) and the C-terminus (c) are shown. The S100A domain, repeats, linkers and a conserved sequence motif close to the carboxy-terminus (C-motif) are indicated above the alignments. Amino acid residues, which have been conserved in all species, are shown in red. An alignment of the complete amino acid sequences is shown in Fig. S3. (d) Amino acid sequence alignment of the carboxy-terminal region of dolphin filaggrin and other S100 fused-type proteins (SFTPs). ‘//’ indicates sequence gaps that were introduced to facilitate the depiction of all sequence ends. The sequence motif of trichohyalin that was investigated by Takase and Hirai (41) is underlined. The consensus sequence of the ancestral carboxy-terminal motif of SFTPs is shown below the alignment. Residues of this motif are indicated by yellow shading in individual SFTPs. In panels a–d, red fonts indicate amino acid residues that are identical in all sequences aligned.

Homologs of the *FLG* gene were identified in members of the phylogenetic clade of oceanic and river dolphins (40), that is the bottlenose dolphin, the orca and the baiji (Yangtze river dolphin), but not in the sperm whale and the minke whale. The proteins encoded by these genes are homologous to filaggrin of cattle (Fig. S3) and contain a S100 domain (Fig.1a), a region of sequence repeats (Fig.1b) and a characteristic carboxy-terminus (Fig.1c). The number of filaggrin sequence repeats is smaller in dolphins (maximum *n* = 5) than in cattle (*n* = 10) (Fig. S3) and humans (*n* = 10–12). The filaggrin repeats of dolphins and cattle are of similar length, with all being shorter than human filaggrin units (22). The sequences of the linkers between the filaggrin repeats are largely conserved among cetartiodactyls but different from those of human filaggrin (Fig. S4). Furthermore, the amino acid sequences of filaggrin repeats were more similar between dolphins and cattle (70–72%) than between cattle and human (41%) (Table S2).

The alignment of the carboxy-terminal sequences of dolphin filaggrin and SFTPs from phylogenetically distant species of amniotes allowed us to define a sequence motif conserved in representatives of all types of SFTPs (Fig.1d). Notably, this motif with the consensus sequence SPLY(D/E)Y(V/L)QEQ(K/R) overlaps with a carboxy-terminal motif of trichohyalin (Fig.1d, underlined) that is critical for binding to keratins (41).

We could also identify the non-coding exon 1 and the promoter of the *FLG* genes of dolphins. Comparison of the proximal promoter sequences revealed high degrees of sequence conservation including conservation of a putative AP1 binding site (Fig. S5).

Strikingly, all SFTP genes other than *FLG* are deleteriously mutated in the five cetaceans investigated (Table S1). In addition to orthologs of the human SFTPs (cornulin, filaggrin, filaggrin 2, hornerin, repetin, trichohyalin and trichohyalin-like 1), we searched for a trichohyalin-like 2 (*TCHHL2*) gene, which has been reported recently for sheep, opossum, platypus and other mammals (26), in genomes of cetaceans and in the human genome. None of the available cetacean genomes contained intact *TCHHL2* (Table S1). The human genome contained a deleteriously mutated gene remnant corresponding to *TCHHL2* (Fig. S5), indicating inheritance of this gene from a common ancestor of mammals (26) and independent inactivations of this gene in the evolutionary lineages leading to cetaceans and humans. Taken together, our data suggest that, in contrast to terrestrial mammals (24,26), cetaceans have lost SFTP genes with the notable exception of *FLG* in dolphins.

### Caspase-14 has been lost, whereas saspase has been conserved in all cetaceans

**Figure 2:**
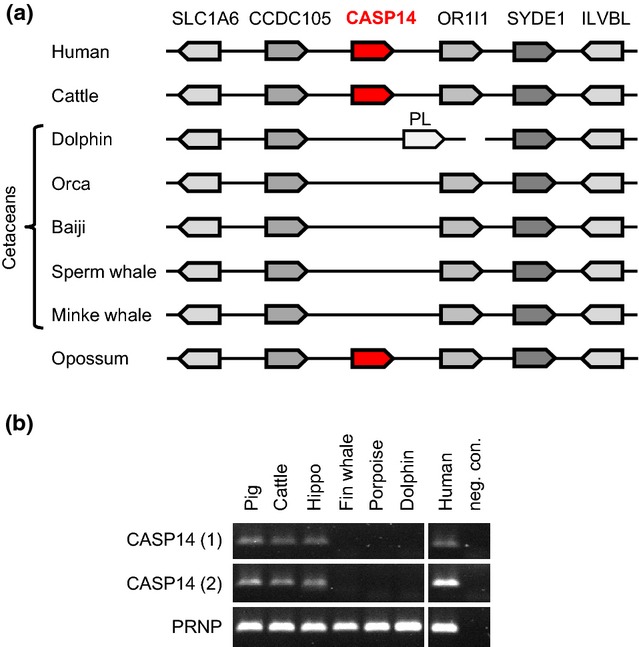
Caspase-14 has been lost in cetaceans. (a) Schematic overview of the *CASP14* locus in cetaceans and other mammals. (b) PCR screening for segments (1 and 2) of the *CASP14* gene in cetaceans (fin whale, porpoise and bottlenose dolphin) and other mammals. Genomic DNA was amplified with primers annealing to conserved sites of *CASP14*. The conserved gene for prion protein (*PRNP*) was amplified to confirm the integrity of all genomic DNAs used.

To determine the presence or absence of caspase-14, we investigated genome sequences and performed PCR screenings with primers that annealed to regions evolutionarily conserved among *CASP14* genes but no other caspase genes. Homologs of caspase-14 were detected neither by BLAST screening of the entire genomes nor by scrutinizing the regions flanked by the conserved genes, which are the neighbours of *CASP14* in the genomes of terrestrial mammals (Fig.2a; Fig. S7). In contrast to the absence of *CASP14*, genes for caspases-15 and 16, which resemble caspase-14 with regard to sequence but not expression pattern (27,39,42,43), were readily identified in the available genome sequences of cetaceans (Fig. S8 and S9). Notably, the *CASP16* gene of all cetaceans was interrupted by an in-frame stop codon.

As the sequence assemblies of cetaceans other than the baiji were not contiguous in the region syntenic with the *CASP14* locus, we performed an additional search for *CASP14* sequences. We designed primers that anneal to conserved *CASP14* sequences of diverse mammals but not to other caspase genes. PCR screening of genomic DNA from terrestrial mammals and cetaceans yielded caspase-14-specific bands in all mammals tested, including the closest terrestrial relative of cetaceans, that is the hippopotamus, but not in the bottlenose dolphin, the harbour porpoise and the fin whale (Fig.2b).

**Figure 3:**
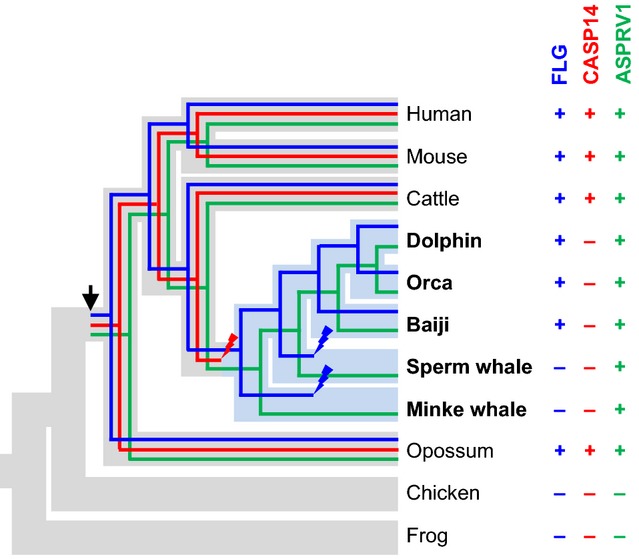
Phylogenetics of filaggrin, caspase-14 and saspase in cetaceans. The presence (+) or absence (−) of genes encoding filaggrin, caspase-14 and saspase was mapped onto a phylogenetic tree (40,58). The phylogenetic trees of the three genes are shown within the phylogenetic tree of the species. Fully aquatic lifestyle is indicated by blue colour of the corresponding branches of the species tree. An arrow indicates the origin of genes, and strike symbols indicate loss of genes. FLG, filaggrin; CASP14, caspase-14; and ASPRV1, aspartic peptidase retroviral-like 1 (saspase).

Saspase is a protease phylogenetically unrelated to caspase-14 but expressed in the same pattern, that is exclusively in the stratum granulosum (44,45). Knockout of the murine *ASPRV1* gene, which encodes saspase, and *in vitro* experiments have suggested that saspase cleaves profilaggrin in the linker regions between filaggrin monomers (17) (Fig. S4). Our comparative genomics analysis suggests that the evolutionary origin of *ASPRV1* – perhaps by insertion of a retroviral gene – coincided with the origin of filaggrin (Fig.3; Fig. S10). A screening for *ASPRV1* in cetaceans identified *ASPRV1* genes encoding apparently functional proteins (Fig. S10). The conservation of *ASPRV1*/saspase in species without filaggrin (that is, in whales) (Fig.3) indicates that saspase has beneficial roles that are unrelated to the processing of profilaggrin.

## Discussion

The results of this study reveal a discrepancy in the pattern of evolutionary fates of filaggrin and two of its presumable proteolytic regulators, saspase and caspase-14. Besides other proteases (46), saspase contributes to the conversion of profilaggrin to filaggrin monomers, and caspase-14 is involved in the breakdown of filaggrin to amino acids (21). Assuming that filaggrin, saspase and caspase-14 strictly depend on these interactions for being functional, the conservation or loss of these three genes would be expected to occur in a concerted manner. However, only in the minke whale and in the sperm whale, filaggrin and caspase-14 have been lost together. By contrast, saspase has been conserved in whales despite loss of filaggrin. Likewise, filaggrin has been conserved, whereas caspase-14 has been lost in dolphins. These patterns of gene evolution indicate that filaggrin, saspase and caspase-14, all of which are expressed specifically in terminally differentiated keratinocytes, are not strictly interdependent.

The expression pattern and the physiological role of filaggrin in dolphins remain to be determined. In preliminary experiments, we could not detect filaggrin by immunohistochemistry (data not shown) in abdominal skin from a stranded dolphin, whereas several bands of unknown identity were detected by immunoblot analysis with an antibody against murine filaggrin (Fig. S11). As filaggrin is not only expressed in the epidermis but also at cornified sites of the oral epithelium in humans (47) and rats (48), filaggrin may be expressed at equivalent sites of the dolphins even if keratohyalin granules are not present there (49). In the epidermis of terrestrial mammals, the expression and the proteolytic processing of filaggrin are controlled by the humidity of the environment (50–52). As a relative humidity of more than 95% suppresses proteolysis of filaggrin (52), filaggrin may not function as a source of amino acids but only as a structural component of cornified keratinocytes in dolphins and in the oral epithelium of terrestrial mammals. In future studies, fresh samples from epidermis as well as from the oral epithelium of dolphins should be collected and investigated with antibodies of confirmed cross-reactivity with dolphin filaggrin.

The absence of keratohyalin granules despite conservation of filaggrin in dolphins (33,49) suggests that post-translational processing and/or transport of filaggrin in dolphins differs from that in humans. Our sequence comparisons show that the filaggrin repeat units and linkers between sequence repeats of dolphins differ significantly from their counterparts in humans (Fig. S3). However, there are also considerable sequence differences between the repeat region of human filaggrin and filaggrins of other terrestrial mammals such as artiodactyls (Fig. S3) and the dog (53). Together with previously published data, the results of this study indicate that the function(s) of profilaggrin require little conservation in the sequence of the filaggrin repeat region.

Besides the S100 domain and the presence of sequence repeats, filaggrin of dolphins contains a conserved sequence motif close to its carboxy-terminus. Our alignment of SFTP amino acid sequences, that has been improved relative to an alignment reported recently (24), shows that proteins of all types of SFTPs (i.e. filaggrin, hornerin, trichohyalin, etc.) have this motif, suggesting that is has been inherited from a common ancestral SFTP gene. The ancestral carboxy-terminal motif of SFTPs is located after the repetitive region and is followed by a protein region, the length of which varies considerably among SFTPs (Fig.1d). Notably, individual SFTPs of some species appear to have lost this motif. For example, filaggrin of the mouse lacks the evolutionarily ancient motif and instead contains a carboxy-terminal stretch of four tyrosinase (Y) residues that are also present in human filaggrin (54) but not in cetartiodactyls (this study, Fig.1d). Importantly, the ancestral carboxy-terminal SFTP sequence motif overlaps with the carboxy-terminal sequence of trichohyalin that has been shown experimentally to be essential for the binding of trichohyalin to keratins (41). This finding suggests that filaggrins of dolphins as well as other SFTPs utilize this motif to interact with keratin filaments. This hypothesis should be tested in future studies because it may be relevant for the effects of protein-truncating human *FLG* mutations in ichthyosis vulgaris and atopic dermatitis.

In contrast to the *FLG* gene, which has been conserved in a subset of cetaceans, all other SFTPs have been inactivated in this clade of mammals. This finding establishes a correlation between the roles of trichohyalin, trichohyalin-like 1 and cornulin in the inner root sheath of the hair (24) and the loss of hair in cetaceans. Likewise, the nails have been lost in cetaceans, obviating the proposed requirement for trichohyalin in the nail unit (24). It also remains to be investigated whether the presence of filaggrin in dolphins and its absence in whales result in phenotypical differences that may be associated with keratin aggregation.

Our data suggest that caspase-14 has been lost in cetaceans. Caspase-14 is a protease specifically expressed in the outermost layers of the epidermis where keratinocytes are converted into corneocytes, the dead building blocks of the skin barrier to the exterior environment, that is the stratum corneum (55). Caspase-14 knockout mice have a disturbed degradation of filaggrin to amino acids and urocanic acid, elevated sensitivity to ultraviolet light-induced DNA damage and increased transepidermal water loss (21,56). Our finding that caspase-14 has been deleted in cetacean species, which have filaggrin, indicates that filaggrin does not strictly depend on neither direct nor indirect processing by caspase-14, at least in water-living mammals. In this regard, it is worth noting that cetaceans need protection against UV radiation (57), which, however, may not be achieved by water-soluble factors such as filaggrin-derived urocanic acid (16).

Besides cetaceans, there are other aquatic mammals that should be investigated with regard to their SFTP genes in future studies. Of particular interest are the sirenians, the mammalian order comprising manatees. In a preliminary screening of the genome of the West Indian manatee (*Trichechus manatus*), we have detected conservation of filaggrin and SFTPs, which may have roles in the hair follicles (our unpublished data). In the manatee, caspase-14 is conserved, whereas *ASPRV1* contains a premature stop codon and several changes of residues that are conserved in other species, indicating that, despite conservation of filaggrin, saspase may be non-functional in this species. Semi-aquatic mammals such as the hippopotamus and pinnipeds (seals, sea lions and walrus) are also likely to have distinct adaptations of their epidermis to the aquatic environment and may be useful in comparisons to terrestrial mammals.

Taken together, this study demonstrates that, during the evolution of cetaceans, multiple SFTPs and caspase-14 have been lost in parallel with changes in the morphology of the epidermis in cetaceans. We propose that the absence of these genes correlates with the loss of distinct functions of the epidermis that are required for survival in a terrestrial environment. Thus, the present study exemplifies how comparative genomics studies can complement targeted gene knockout studies in the mouse by identifying ‘evolutionary gene knockout’ animals. The screening for genes that have been conserved in terrestrial mammals but lost in fully aquatic mammals may be a useful approach to identify or confirm associations of genes with distinct functions in the epidermis.

## Supporting Information

**Figure S1.** Phylogenetic tree of cetaceans and other vertebrates. **Figure S2.** Histology of the bottlenose dolphin skin. **Figure S3.** Alignment of amino acid sequences of filaggrin proteins. **Figure S4.** Amino acid sequence alignment of representative linker regions of filaggrin proteins from different species. **Figure S5.** Nucleotide sequence alignment of the proximal promotor of filaggrin genes. **Figure S6.** The trichohyalin-like 2 (*TCHHL2*) genes of whales and humans contain deleterious mutations. **Figure S7.** Amino acid sequence alignment of capase-14 proteins. **Figure S8.** Amino acid sequence alignment of caspase-15 proteins. **Figure S9.** Nucleotide sequence alignment of exon 6 of the *CASP16* gene of various species. **Figure S10.** Conservation of *ASPRV1* (saspase) in cetaceans. **Figure S11.** Immunoblot analysis of epidermal protein extracts using an anti-filaggrin antibody. **Table S1.** S100 fused-type protein (SFTP) genes of cetaceans. **Table S2.** Interspecies amino acid sequence identity (%) of filaggrin repeats and linkers.
