# Supplementary material for: Comparative genomics reveals conservation of filaggrin and loss of caspase-14 in dolphins
Source: Exp Dermatol. 2015 Apr 27;24(5):365–9. doi: 10.1111/exd.12681 (PMC4437054; doi:10.1111/exd.12681)
Supplement: Supplementary file 1 [file exd0024-0365-sd1.pdf]

Supplementary Data

Comparative genomics reveals conservation of filaggrin and loss of caspase-14 in dolphins

Bettina Strasser, Veronika Mlitz, Heinz Fischer, Erwin Tschachler, Leopold Eckhart

Contents

Supplementary Figures S1 – S11

Supplementary Tables S1 – S2

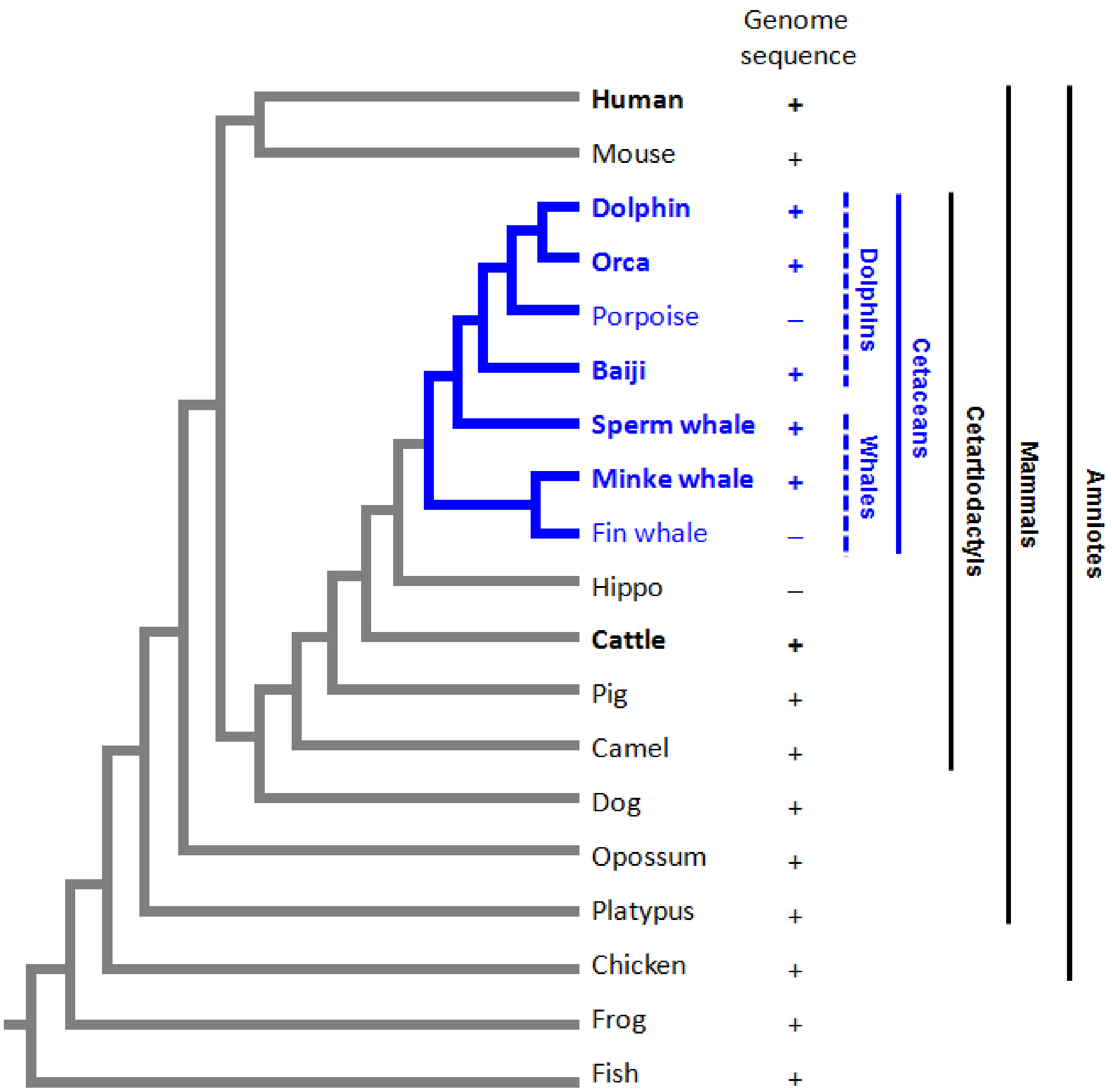

Suppl. Fig. S1. Phylogenetic tree of cetaceans and other vertebrates. The phylogenetic tree shows the relationship of the species mentioned in this study. Phylogenetic clades are indicated on the right. The species referred to as "dolphin" is the bottlenose dolphin (*Tursiops truncatus*). The clade referred to as "dolphins" comprises the bottlenose dolphin and orca (oceanic dolphins) and baiji (river dolphin). The order of cetaceans is highlighted by blue color. The availability or absence of genome sequences in the GenBank database is indicated by + and –, respectively. The comparative genomics studies were focused on the species indicated with bold letters.

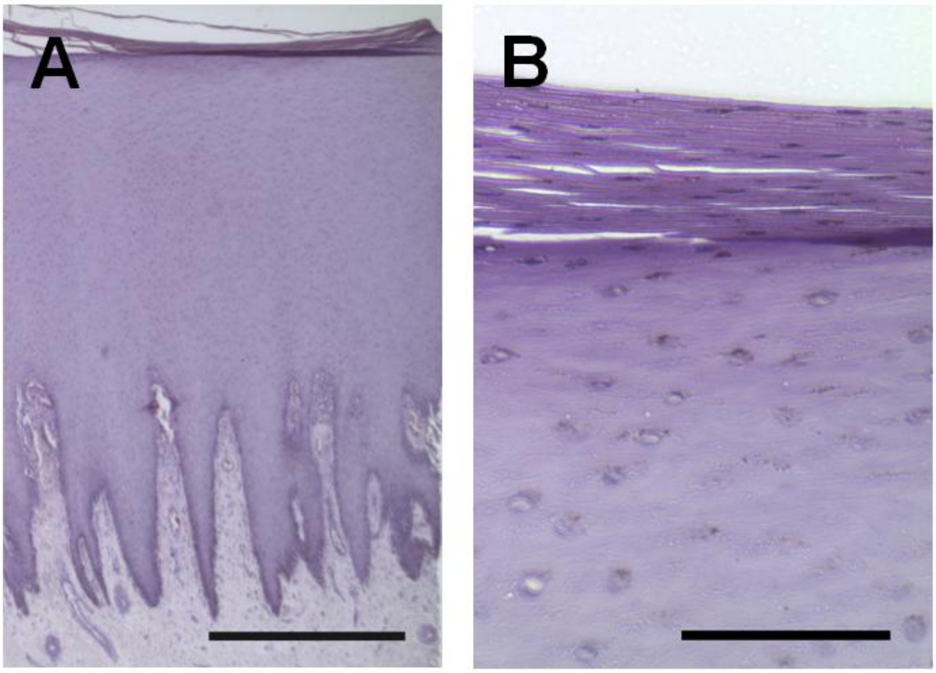

Suppl. Fig. S2. Histology of the bottlenose dolphin skin. Skin sections were stained with hematoxylin and eosin. (**A**) Section of the skin. The epidermis is several millimeters thick. Bar, 1000 µm. (**B**) Outermost part of the epidermis. Panels A and B are from different sections. Bar, 100 µm.

```
        S100 domain
Dolphin MSTLLENITAIIKLFHEYSKTDKETDTLSAKELKELLEAEFQPILKNPDDPDTADVFMHILDVDHNHKIDFTEFFLMVFKLAQAYY-YTQRPNFKTLGKKQKKNRY    105
   Orca MSTLLENITAIIKLFHEYSKTDKETDTLSAKELKELLEAEFQPILKNPDNPDTADVFMHILDVDHNHKIDFTEFFLMVFKLAQAYY-YTQRPNFKTLGKKQKKNRY    105
  Baiji MSTLLENITAIIKLFHEYSKTDKETDTLSAKELKELLEAEFQPILKNPDDPDTADVFMHILDVDHNNKIDFTEFFLMVFKLAQAYY-YTQRPNFKTLGKKQEKYRY    105
 Cattle MSTLLENINDIIKIFHKYSKTDKETDTLSEKELKELVEVEFRPILKNPGDPDTAEVFMYNLDRDHNNKIDFTEFFLMVFKVAQVYYSYTQRQNLQRAGQKQKKCTY    106

Dolphin HYE---DDTEEEGKEERERKSSHS-RSDGKKEDRTEEEEE-GRSRHGSSSG-REGRHR-------NRSGKKRHESSREKKRRASSTELKERSHMSSVSPIREYEGK    198
   Orca HYE---DDTEEEGKEERERKSSHS-RSDGKKEDRTEEEEE-GRSRHGSSSG-REGRHR-------NRSGKKRHESSREKKRRASSTELKERSHMSSVSPIREYEGK    198
  Baiji HYE---DDTEEEGKEERERKSSHSGRSDGKKKDRTESPRGRGRSRHGSSSGREGRRGDRATSGHRNRCGKKHHESSREKKRRASSTELKERSHMSSVSPTREYEGK    208
 Cattle HYGDEEDDTEED-KEETERKYSHS-RSDGKTQDRSKSPRGRGKKRHGSKSGSKQRRGDTPTSGLRHGCSKK-HESRREKKRRPSSTEPKERRHMSSVSPTRGYEEK    209

                                                    partial repeat                                 ------ linker -----
Dolphin EEERGYENKDRGCEKWIGSEPKGSYQVCEETVTMDFQSGCSTQQVSSISKGSDSKEHSQDSGRQPVITHGRSPSSSRNQHGSSHDQSGDSPKHSESHQGRTDTHRKSES 307
   Orca EEERGYENKDRGCEKWIGSEPKGSYQVCEETVTMDFQSGCSTQQVSSISKGSDSKEHSQDSGRQPVITHGRSPSSSRNQHGSSHDQSGDSPKHSESHQGRTDTHRKSES 307
  Baiji EE-RGYENKSRGCEKWIGSERKGLYQVCEETVTMDFQSGCSTHRVSSISKGSNSKEHSQDSGRQPVITHGRSPSSSRNQHGSSHDQSGDSPKHSESHQGRTDTHRKSES 316
 Cattle EEEHGYENKGKTSAKCIGSEYDDSYQVCEDKVTTNFQPSHSKNYGSNITKGRDTEGHSRDTGRKSVFTHARSGSSSRNQNGSVQTHTGDNSTHSESQQE-TN----SES 313

        repeat 1                                                                                   ------ linker -----
Dolphin VHGKSGSSTTQRQGRHHEQEKDSSRPSGTGHGHASTGSGSSRHRESSVGQSSDSEIQSGNSGRYSVTTHGRSGSSSKNQHGSSQGQSRDSSRHSESHQGRTDTHKKSES 416
   Orca VHGKSGSSTTQRQGRHHEQEKDSSRPSGTGHGHASTGSGSSRHRESSVGQSSDSKIQSGNSGRYSVTTHGRSGSSSKNQHGSSQGQSGDSSRHSESHQGRTDTHKKSES 416
  Baiji VHGKSGSSATQRQGHDHEQEEDSSRPSGTGHGHASTGSGSSSHRESSVGQSSDSEIQSGDSGRYSVTTHGRSGSSSKNQHGSSHGQSGDSSRHSELHQGRTDTHRKSES 425
 Cattle VHRRS-RNTGQRQGSHHEQSRDSSRHSGTRHGQPSTGSGGSRHRESSVSQASDSEGHSEDSGRQSVTTHGRPGSSSRNQHGSSQGQTGDSSRHSESHQGRH-----SDS 416

        repeat 2                                                                                   ------ linker -----
Dolphin GHSKSGSSTTQRQGHHHEQERDSSRHSGTGHGHTSDGSRSGKDRESSVGQSSDSEGQSEDSHRHSVTTHGRSGSSSRNQHGSSHGRSGDSSRHSESHQGRTDTHRKSES 525
   Orca GHSKSGSSTTQRQGHHHEQERDSSRHSGTGHGHTSDGSRSGKDRESSVGQSSDSEGQSEDSDRHSVTTHGRSGSSSRNQHGSSHGRSGDSSRHSESHQGRTDTHRKSES 525
  Baiji VHGRSGSSTTQRQGLHHEQERDSSRYSGTGHGHTSDESRSGKDRKSSVGQSSDSEGQSEDSGRHSVTTHGRSGSSSRNQHGSSHGWSGDSSRHSESHQGRS-AYRKSES 533
 Cattle LQRRSGTSTGQRQGSHHEQSRDSSRHSGTQQGQTSTGSGSSRHRDSSVSQASDSEGHSEDSGRQSVTTHGRPGSSSRNQHGSSHGQTGDSSRHSESHQ-RSH----SEA 520

        repeat 3 + partial repeat                                                                  ------ linker -----
Dolphin VHGESGSSTKQRQGHHHEQEKDNSRHSGIGHGHASTGSRKSKQRESSVGQSSDSEGQSEDSGRHSVTTHGRSGSRSRNQHGSSHGWSGDSSRHSESHQGRS-AYRKSES  633
   Orca VHGESGSSTKQRQGHHHEQKKDSSRHSGIGHGHTSTGSRKSRQRESSVGQSSDSEXXXXXXXXXXXXXXXXXXXXXXXXXXXXXXXXXXXXXXXXXXXXXXXXXXXXXX  634
  Baiji ---------------------------------------------------------------------------------------------------------------
 Cattle VHERSGSSTGQRQRSHHEQSRDSSRHSGTGHGQISAESGNGRHRESSVSQASDSEGHARDSGRHSETTHGRYGSSSRNQHGSSHGQTGDSYRHSETNQGRHNKR-----  624
 Cattle -------------------------------------------------------HTRDSGRHSETTHGRPGSSSRNQHGSSHGQSGDSSRHSESHQERH-----SES  672

        repeat 4                                                                                   ------ linker -----
Dolphin VHSKSGSSTTQRQGHHHEQERDSSRYSGTGHGHTSTGSGSNRHRESSVGWSSDSEGQSGDSGRHSVTTHGGSGSSSRNQHGSSHGWSGDSSRHSESHQGRS-AYRKSES  741
   Orca XXXXXXXXXXXXXXXXXXXXXXXXXXXXXXXXXXXXXXXXXXXXXXXXXXXXXXXXXXXXXXXXXXXXXXXXXXXXXXXXXXXXXXXXXXXXXXXXXXXXXXXXXXXXXXX  743
  Baiji GHRKSGSSTTQRQGHHHEQERDSSRHSGTGHGHTSTGTGSNRHRESSVGWSSDGEGQSGDSGRHSVTTHGRSGSSSRNQHGSSHGQSRDSSRHSESHQGRTDTHRKSES  642
 Cattle VHGRSGSSTGQRQGSHHEQSRDSSRHSGTRHGQTSAESGNGRHRESSVSQASDSEGHTRDSGRHSETTHGRPGSSSRNQHGSSQGQTGDSSRYSAYHQGRH-----SES  776

        repeat 5 + partial repeat                                                                  ------ linker -----
Dolphin GHSKSGSSTTQRQGHHHEQERDSSRYSGTGHGHTSTGSGSNRHRESSVGWSSDGEGQSGDSSRHSVTTHGRSGSSSRNQHGSSHGQSGDSSRHSESHQGRTDTHKKSES  850
   Orca XXXXXXXXXXXXXXXXXXXXXXXXXXXXXXXXXXXXXXXXXXXXXXXXXXXXXXXXXXXXXXXXXXXXXXXXXXXXXXXXXXXXXXXXXXXPESHQGRTDTHKKSES  852
  Baiji VHGESGSSTKQRQGCHHEQERDNSRHSGIGHGHASTGSRKSRQRESSVGQSSDSEGQSEDSGRHSVTTHGRSGFSSRNQHGSSHGRSGDSSRHSESHQGRS-AYRKSES  750
 Cattle VHERSASRTGQRQGSHHEQSRDSSRQSGTGHGHTSAGSGNGRHRESSVSQSSDREGHARDSGRQSVTTHGRPGSSSRNQHVSSHGQIGDSSSHSETNQGSH-----NER  880
 Cattle -------------------------------------------------------HTRDSGRHSETTHGRLGSSSRNQHGSVHGQTRDSPRHSESHQERH-----SES  928

        repeats 6-10 + partial repeat                                                             ------ linker -----
 Cattle VHGRSGSSTGQRQESHHEQSRDSSRQSGTGHGHTSAGSGNSRHRESSVSQASDSEGHSEDSGRQNVTTHGRPGSSSRNQHGSSQGQTGDSSRYSAYHQGRH-----SES 1032
 Cattle VHERSASRTGQRQGSHHEQSRDSSRQSGTGHGHASAGSGINRHRESSVSQASDREGHARDSGRQSVTTHGRPGSSSRNQHVSSHGQIGDSSSHSETNQGSH-----NER 1136
 Cattle -------------------------------------------------------HTRDSGRHSETTHGRLGSSSRNQHGSVHGQTRDSPRHSESHQERH-----SES 1184
 Cattle VHERSGSSTGQRQGSHHEQSRDSSRHSGTRHGQTSTVSGGSRHREPSVSQASDSEGHSEDSGRQSVTTHGRPGSTSRNQHGSVHGQTRDSPRHSESHQERH-----SES 1288
 Cattle VHQRSGSSTGQRQGSHHEQSRDSSRHSGTGHGQTSAGSGSSRHRESSVSQASDSEGHSEDSGRQNVTTHGRPGSSSRNQHGSSQGQTGDSSRYSASHQGRH-----SES 1392
 Cattle VHERSTSRTGQRQGSHHEQSRDSSRQSGTGHGHASAGSGINRHRESSVSQASDSEGHSEDSGRQNVTTHGRPGSSSSNQHGSSHGQTGDSSRHSESHQGSH-----GES 1496

        partial repeat                                                               C-motif
Dolphin GHSKSGSSTTQRQGHHHEQERDSSRHSGTRHGHTSTISGSGRHKESNISQASNTEGHSGDSGRQPLTTQGWSAFYSRNQSHGSDQGWRHGSYGSADYDYGQSGFGHSQD   959
   Orca GHSKSGSSTTQRQGHHDEQERDSSRHSGTGHGHTSTISGSGRHKESNISQDSNTEGHSGDSGRQPLTTQGCSAFYSRNQSHGSDQGWRHGSYGSADYDYGQSGFGHSQD   961
  Baiji GHRKSGSSTTQRQGHHHEQERDSSRHSGTEHGHTSTISGSGRHKESNISQASNTEGHSGDSGRQHLTTQGWSAFYSRNQSHGSDQGWRHGSYGSEDYDYGQSGFGHSQD   859
 Cattle VHQRSGSSTGQRQGSHHEQSRDSSRHSGTGHGETSTGSGSSRHRESSVSQSSDSKGHSRGSGNQTVTNQRMSAFYSRFQNHGAGQVWRHGSYGSSNYDYGQLGTGHSPD  1605

Dolphin GSVSHDSSHMGARDRFEYR---------------SIYG-IQY-NRQ-----  988
   Orca GSVSHDSSHMGARDRFEYR---------------SIYG-IQY-NRQ-----  990
  Baiji GSVSHDSSHMGARDRSEYR---------------SIYG-IQY-NRQ-----  888
 Cattle ENFSHDSSHVEKRDKPEYRGELMRSNITVRNIHPGTYGHSNYISKQLGFGQ 1656
```

Suppl. Fig. S3. Alignment of amino acid sequences of filaggrin proteins. Amino acid sequences of filaggrin proteins of bottlenose dolphin (*Tursiops truncatus*), orca (*Orcinus orca*), baiji (*Lipotes vexillifer*) and cattle (*Bos taurus*) were aligned. The S100A domain, sequence repeats, linker segments and a conserved sequence motif close to the C-terminus (C-motif) are indicated above the alignment. Red fonts indicate amino acid residues, that have been conserved in all species. Note that the sequence of cattle filaggrin contains more sequence repeats and partial repeats than filaggrin proteins of other species do. The sequences of several repeats of the cattle are written without alignment to counterparts of cetacean filaggrins. Yellow shading highlights amino acid residues conserved among different sequence repeats. Numbers of the last amino acid residue are shown on the right. Fig. 1A-C show segments of this alignment.

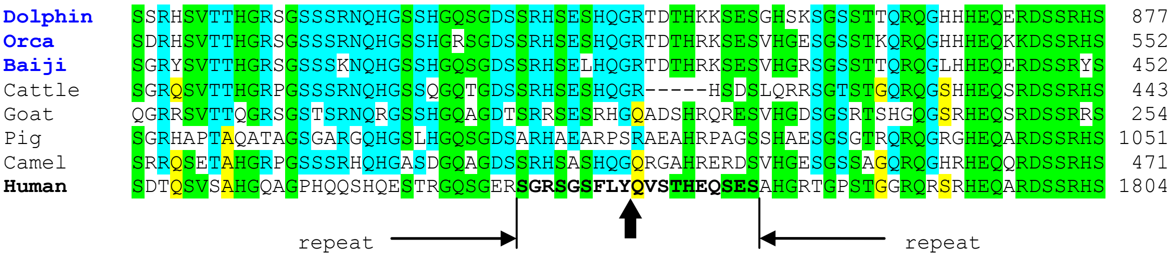

Suppl. Fig. S4. Amino acid sequence alignment of representative linker regions of filaggrin proteins from different species. Partial amino acid sequences of human profilaggrin and profilaggrin proteins of species from the mammalian order Cetartiodactyla (comprised of cetaceans and artiodactyla) are aligned. The borders between the linker peptide and the flanking filaggrin monomer repeats (22) are indicated in the human sequence. The vertical arrow indicates the site of human profilaggrin cleavage by saspase (17). Amino acid residues identities are indicated by color shading. Yellow shading indicates residues that are conserved in humans and at least 2 other species but not in cetaceans. Blue shading indicates residues that are conserved in at least 2 cetaceans and at least 2 other species but not in humans. Green shading indicates residues that are conserved in humans and cetaceans. The number of the last amino acid residue of each sequence is shown on the right. Genbank accession numbers: cattle filaggrin, XP_001255583.5; goat filaggrin, XP_005677634.1; pig filaggrin, XP_005663493.1; camel filaggrin, XP_006188973.1; human filaggrin, NP_002007.1. The sequences of filaggrins of the dolphin, orca and baiji are shown in Suppl. Figure S3.

```
Dolphin  CATAAAA-TCTCCAGTAT--ATGAGAATAATGTAAAGCGAAATTTGGAACTTGGCAAGACAAGTACAGATGAGGACATGAAGAAGTTGGGAAGCAAACAC
   Orca  CATAAAA-TCTCCAGTAT--ATGAGAATAATGTAAAGCTAAATTTGGAACTTGGCAAGACAAGTACAGATGAGGACATGAAGAAGTTGGGAAGCAAACAC
  Baiji  CATAAAA-TCTCCAGTAT--ATGAGAATAATGTAAAGCTAAATTTGGAACTTGGCAAGACAAGTACAGATGAGGACATGAAGAAGTTGGGAAGCAAACAC
 Cattle  CATGAAA-GCTACAACTTGCACGAGAATGATGTAAACCTAAACT-GTAATTTGGTAAAACTGGTACAGATGAGTACGTGAGCAAGTTGGGAAGTAAACAG
    Pig  CATAAAAATCACCAATAT--ATGGGAATGATGTAAACCTACATTTGGAACTTAGTAAAACAAGTACAGATGAGTACGTGAGGAAGTTGGGAAGTAAACAC
  Camel  CATAAAA-TCCCCAATAT--ATGGGAATGATGTAAACTTAAATTTGGAACTTGGTAAAACAAGTACAGATGAGTACGTGAGCGATTTGGGAAGTAAACAC
  Human  CATAAAA-TTTCCAAT-------------ATGTAAACCCAAATTTGGAACTTGCTGAAACAAGTACAGATGAGTACGTGAGGAAGCTGGGAAGTAAACAC

                                                          AP1
Dolphin  A--TTGCTAGAGAAATAATAGAGGTGGAGATATGGGTGGATCTGGGTTTGGTTAGGAATGAATCAGACCAACCTACAGAAGA---TGGCTCCTCCCTGGA
   Orca  A--TTGCTAGAGAAATAATAGAGGTGGAGATATGGGTGGATCTGGGTTTGGTTAGGAATGAATCAGACCAACCTACAGAAGA---TGGCTCCTCCCTGGA
  Baiji  A--TTGCTAGAGAAATAATAGAGGTGGAGATATGGGTGGATCTGGGTTTGGTTAGGAATGAATCAGACCAACCTACAGAAGA---TGGCTCCTCCCTGGA
 Cattle  G--TCGCTAGAGAAATA---GAGGTGGAGATATGGGTGGATCTGAGTTTGGTTAGGAATGAATCAGTCCATCCTACAGAGGA---TGGCTCCTCCCTGGA
    Pig  A--TAGCTACAGAAATA---GAGGTGGAGATATGGGTGGACCTGGGTTTGGTTAGGAATGAATCAGTCCATCCTACAGAGGAGGATGGCTCCTCCCTGGA
  Camel  A--TTGCTAGAGAAATA---GAGGTGGAGATATGGGTGGATCTGGGTTTGGTTAGGAATGAATCAGACCATCCTACAGAGTA---TGGCTCCTCCCTAAA
  Human  AGGTTGCTGGAGAAATA---GAGGTGGAGATATGGGTGGATCTAGGTTTGGTTAGGAATGAATCAGACCATCCCACAGAGGG---TGGCTCCTCCCTGCA

                   TATA box
Dolphin  TGGA-CCTGCTATAAAAAGGCCATTATCCCAGACTTCAGCACACAGCAAGCTCCTTCAGGCTACATTCTACTTGCTCTTCTGGTGAAGGAAGGAAGTAAG
   Orca  TGGA-CCTGCTATAAAAAGGCCACTATCCCAGACTTCAGCACACAGCAAGCTCCTTCAGGCTACATTCTACTTGCTCTTCTGGTGAAGGAAGGAAGTAAG
  Baiji  TGGA-CCTGCTATAAAAAGGCCATTATCCCAGACTTCAGCACACAGCAAGCTCCTTCAGGCTACATTCTACTTGCTCTTCTGGTGAAGGAAGGAAGTAAG
 Cattle  TGGA-CCTGCTATAAAAAGGCCATGATCCCAGTCTTCAGTACCCAGCAAACTCCTCCGGGCTACAATCTCCTTGCTCTTCTGGTGAAGGAAGGAAGTAAG
    Pig  TGGA-CCTGCTATAAAAAGGCCATTATCCCAGACTTCAGCACCCAGCAAGCTCCTTCAGGCTACATTTCCTTGCTCTTCTGGTGAA----GGAAGTAAG
  Camel  TG-A-CCTGCTATAAAAAGGCCATTATTCCAGGCTTCAGCACCCAGCAAGCTCCTTCAGGCTACGTTCTACTTGCTCTTCTGGTGAA----GGAAGTAAG
  Human  TGGGGCCTGCTATAAAAGGGCCATTATCTCAGCCTTCAGTACCCAGCAGGCTCCTTCAGGCTACATTCTATTTGCTCTTTTGGTGAA----CAAGGTAAG
```

Suppl. Fig. S5. Nucleotide sequence alignment of the proximal promotor of filaggrin genes. Nucleotides conserved in all species are highlighted by red color. The putative binding sites of the transcription factor AP1 and the TATA box are indicated by colored frames. The GT splice donor site at the start of intron 1 is underlined.

A

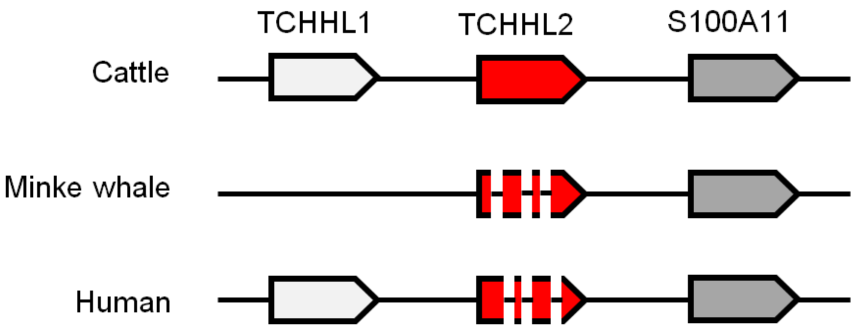

B

```
                 M  T  H  R  L  L  R  S  I  I  S  I  I  D  A  F  L  P  N  A  K  S  D  G
      Cattle    ATGACTCACCGTCTTCTGAGAAGCATCATCAGTATCATTGATGCTTTCCTCCCTAATGCAAAGAGTGATGGA
 Minke whale    ATGCCTCACTGTCTTCTGAGAAGTGTTATCAGTATCATCAATGTTTTCCACTCTTATGCAAAGAGTGATGGA
       Human    ATGCTTCCCTATCTTCTGAGGAGCAGCATCAGTATCATCGACATTTTCCACAAGCACGCA--GAGTGATGGA

                 D  C  Q  S  L  N  K  T  E  L  K  K  L  L  Q  E  E  F  G  N  A  L  E
      Cattle    GACTGTCAGAGCCTGAACAAGACAGAACTTAAGAAACTTCTCCAAGAAGAGTTTGGAAATGCTCTGGAGGT
 Minke whale    GACTGTCAGAGGCTGAACAAGACAGAATTGAAGAAACTTCTCCAACAAGAGTTTGGAAATGCTCCGGAGGT
       Human    GACTGGCAGAGGCTGAACAAGACAAAACTCAAGACACTTCTCAGACAAGAGTTTGGAATGGCCTTGGAGGT
```

C

```
                   E  S  N  N  S  E  T  T  G  K  I  L  Q  Q  L  D  Q  D  G  D  Q  T  I
      Cattle    AGGAATCAAATAATTCTGAGACAACAGGAAAAATCCTCCAGCAGCTGGATCAAGATGGTGACCAAACAATT
 Minke whale    AGAAATCAAGTAATTCTGAGACAACATAAAAAATCCTCCAACAGCTGGATCAAGATGGTGACAAAACAGTT
       Human    AGAAAACAACTAATTCTGAGACAATAG-AAAAATCCTCCAACAGCTGGATCAAGATGGTCACAAAACAGTT

                 D  F  S  E  L  I  L  L  M  F  A  V  T  T  A  Y  Y  A  H  I  K  P  L  L
      Cattle    GATTTCAGTGAATTAATTTTGCTCATGTTTGCAGTGACAACAGCCTACTATGCCCACATAAAACCTCTTCTC
 Minke whale    GATTTCAGTGAATTCATTTTGCTTGTGTTCGGAGTGACAAAAACCTATCATGCAAGCATAAAACCTCTTCTC
       Human    GATTTCAGTGAATTCATTTTGCTGGTGCTTACAGTGACAAAAGCCTATCATGCATGCATAAAAGCTCTTCTC
```

Suppl. Fig. S6. The trichohyalin-like 2 (*TCHHL2*) genes of whales and humans contain deleterious mutations. (**A**) Schematic depiction of the *TCHHL2* gene locus of the cattle (*Bos taurus*), minke whale (*Balaenoptera acutorostrata scammoni*) and human (*Homo sapiens*). The direction of the genes is indicated. The *TCHHL2* genes of the minke whale and human are interrupted by in-frame stop codons and frame shift mutations. (**B**, **C**) Nucleotide sequence alignments of the segments of the *TCHHL2* gene. The conceptual translation of the cattle sequence is shown above the nucleotide sequences. (**B**) Alignment of sequences homologous to the coding sequence within exon 2 of cattle *TCHHL2*. (**C**) Alignment of sequences homologous to the start of exon 3 of cattle *TCHHL2*. Nucleotides conserved among the three species are highlighted by red letters. Nucleotide deletions causing reading frame shifts as well as an in-frame stop codon are highlighted by black shading and white font. Splice donor (GT) and acceptor sites (AG) at the ends of introns are underlined. The sequences of the deleteriously mutated remnant of the human *TCHHL2* gene correspond to the nucleotide regions 152067724-152067586 and 152063663-152063523 of human genomic DNA (GenBank accession number NC_000001.11). Accession number for the amino acid sequence of the trichohyalin-like 2 protein in cattle: XP_005197796.1. The location of the *TCHHL2* gene of the minke whale is indicated in Suppl. Table S1.

```
Human  MSNPRSLEEEKYDMSGARLALILCVTKAREGSEEDLDALEHMFRQLRFESTMKRDPTAEQFQEELEKFQQAIDSR
Mouse  MSDPQPLQEERYDMSGARLALTLCVTKAREGSEVDMEALERMFRYLKFESTMKRDPTAQQFLEELDEFQQTIDNW
Cattle MSSPQPLEEETYDMSGARLALTLCVTKAREGSEADLDALERMFQQLGFESTMKRDPTAQQFQEELEKFQQAIDAR

Human  EDPVSCAFVVLMAHGREGFLKGEDGEMVKLENLFEALNNKNCQALRAKPKVYIIQACRGEQRDPGETVG------
Mouse  EEPVSCAFVVLMAHGEEGLLKGEDEKMVRLEDLFEVLNNKNCKALRGKPKVYIIQACRGEHRDPGEELRGNEELG
Cattle EDFVSCAFVVLMAHGLEGRLKGKDEKMVELEDLFQALNNKNCRALRAKPKVYIVQACRGEQRDPGEPVT------

Human  ------GDEIVMVIKDSPQTIPTYTDALHVYSTVEGYIAYRHDQKGSCFIQTLVDVFTKRKGHILELLTEVTRRM
Mouse  GDEELGGDE-VAVLKNNPQSIPTYTDTLHIYSTVEGYLSYRHDEKGSGFIQTLTDVFIHKKGSILELTEEITRLM
Cattle ------GGHLVMITENTPETIPTYTDTLHVFSTIEGYIAYRHDQEGSYFIQTLVDVFINKKGPILELLTEVTRRM

Human  AEAELVQEGKARKTNPEIQSTLRKRLYLQ
Mouse  ANTEVMQEGKPRKVNPEVQSTLRKKLYLQ
Cattle AEAEMVQEGEAKKVNPEIQSTLRKQLYLQ
```

Suppl. Fig. S7. Amino acid sequence alignment of capase-14 proteins. The amino acid sequences of caspase-14 of human, mouse and cattle were aligned. Caspase-14 is absent in cetaceans. Amino acid residues conserved in all species are shown in red color. The cysteine residue in the catalytic center is highlighted by yellow shading. Amino acid residues after which the human caspase-14 proenzyme is proteolytically processed (Hibino T, Fujita E, Tsuji Y, Nakanishi J, Iwaki H, Katagiri C, Momoi T. 2010. Purification and characterization of active caspase-14 from human epidermis and development of the cleavage site-directed antibody. *J Cell Biochem* 109: 487-497) are indicated by blue shading. The latter residues are partly conserved in the mouse but not in the cattle. Accession numbers: human caspase-14, NP_036246.1; mouse caspase-14, NP_033939.1; cattle caspase-14, NP_001192772.1.

```
    Dolphin MEGSPGIGGRSGS------PKIPPAMEELQSRLQETLDLLSSQELRSFRDHLKKVEPRVSQVKLELEGRSPSGLA
       Orca MEGSPGIGGRSGS------PKIPPAMEELQSRLQETLDLLSSQELRSFRDHLKKVEPRVSQVKLELEGRSPSGLA
      Baiji MEGSPGIGWRSGSDSSRAPPKIPPAMEELQSRLQETLDLLSSQELRSFRDHLKKVEPRVSQVKLELEGRSPSGLA
Sperm whale MEGSPGIGWRSGADSSRTPPKIPPAMEELQSRLQETLDLLSSQELRSFRDHLKKVEPRVSQVKLELEGCSPSGLA
Minke whale MEGSPGIGWRSGSDGSRAPPKIPPAMEELQSRLQETLDLLSSQELRSFRDHLRKVEPRVSQVKLELEGRSPSELA
     Cattle MEGSLGSGRRSGSDGQPAPPKISPTTEELQSRLQETLDLLSSQELRSFRDHLKKVEPPVSQVKLELEGHSPSGLA

    Dolphin RLLAKQYYPPAAAKRVLVQVLEQLPRADLLPRWQSAAADGPVIPRKIPKRSYDCVEGELDRYDLSGRRKAFLMCV
       Orca RLLAKQYYPPAAAKRVLVQVLEQLPRADLLPRWQSAAADGPVIPRKIPKRSYDCVEGELDRYDLSGRRKAFLMCV
      Baiji RLLAKQYYPPAAAKRVLVQVLEQLPRADLLPRWQSAAADGPVIPRKIPKRSYDCVEGELDRYDLSGRRKAFLMCV
Sperm whale RLLAKQYYPPAAAKRVLVQVLEQLPRADLLPRWQSAAADGPVIPRKIPKRSYDCVEGELDRYDLSGRRKAFLMCV
Minke whale RLLAKQYYPPAAAKRVLVQVLEQLPRADLLPRWQSAXXXXXVIPRKIPKRSYDCVEGELDHYDLSGRRKAFLMCV
     Cattle KLLAKHYYPAAVAKRVLVQVLELLPRADLLPRWQSAPTDCPVIPRKSLKRSYDCVDGELYRYDLSGRRKAFLMCV


    Dolphin MRDRLGAHQDVLLMEDWLGQCQFENTLCIDPDKMELLGKITSFRDGLNEIKDDIGCCLVALMSHGEEGFIKMKDG
       Orca TRDRLGAHQDVLLMEDWLGQCQFENTLCIDPDKMELLGKITSFRDGLNEIKDDIGCCLVALMSHGEEGFIKMKDG
      Baiji KRNRLGAHQDVLLMEDWLGQCQFENTLCIDPDKMELLGKITSFRDGLNEIKDDIGCCLVALMSHGEEGFIKMKDG
Sperm whale KRNRPGAHQDVLLMEDWLGQCQFENTLCIDPDKMELLGKITSFRDGLNEIKDDIGCCLVALMAHGEEGFIRMKDG
Minke whale KRNRLGAHQDVLLMEDWLGQCQFENTLCIDPDKMELLGKITSFRDGLNEIKDDIGCCLVALMSHGEEGFIRMKDG
     Cattle KKNRLGAHQDVQLMKDWLKECKFEPTLCIDPDKMDLLGKITSFRDELNEIKDDIGCCLVTLMSHGEEGFIKMKDG

    Dolphin EKVSLEGIFEMFNNKNCPALQEKPKIFIIQACRGERRDSGVETDDEPMDSDDGSEKKRLPTFSDYFIIYPTQADH
       Orca EKVSLEGIFEMFNNKNCPALQEKPKIFIIQACRGERRDSGVETDDEPMDSDDGSEKKRLPTFSDYFIIYPTQADH
      Baiji EKVSLEGIFEMFNNKNCPALQEKPKIFIIQACRGERRDSGVETDDEPMDSDDGSEKRRLPTFSDYFIIYPTQADH
Sperm whale EKVSLEGIFEMFNNKNCPALQEKPKIFIIQACRGERKDSGVETDDEPMDSDDGSEKRRLPTFSDYFIIYPTQADH
Minke whale EKVSLEGIFEMFNNKNCPALQEKPKIFIIQACRGERRDSGVETDDEPMDSDDGSEKRRLPTFSDYFIIYPTQADH
     Cattle EKVSLEDIFEMFNNKNCPALQEKPKIFIIQACRGERRDSGVETDDEPMDLDDGSEKKRLPTFSDYFIVYPTQADH

    Dolphin VALRDPRTGSVMIKAMTEVFKQYGNKWHIADFFTKVNNRVVHREFNLRNEPIKVSLVMESTLTKFVYF
       Orca VALRDPRTGSVMIKAMTEVFKQYGNKWHIADFFTKVNNRVVHREFNLRNEPIKVSLVMESTLTKFVYF
      Baiji VALRDPRTGSVMIKAMTEVFKQYGNKWHIADFFTKVNNRVVHREFNLRNEPIKVSLVMESTLTKFVYF
Sperm whale VALRDPRTGSVMIKAMTEVFKQYGNKWHIADFFTKVNNRVVHREFNLRNEPIKVSLVMESTLTKFVYF
Minke whale VALRDPRTGSVMIKAMTEVFKQYGNKWHIADFFTKVNNRVVHREFNLRNEPIKVSLVMESTLTKFVYF
     Cattle VALRDPRTGSVMIKEMTEVFKQYGNKWHLADFFTIVNNRVVHRDFNLCNKPVKVSLVMESTLTKFVYF
```

Suppl. Fig. S8. Amino acid sequence alignment of caspase-15 proteins. The amino acid sequences of caspase-15 from cetaceans and cattle were aligned. Amino acid residues conserved in all species are shown in red color. Note that the gene encoding caspase-15 has been lost during the evolution of the human genome (39). The cysteine residue in the catalytic center is highlighted by yellow shading, aspartic acid residues after which pro-caspase-15 is cleaved by autoprocessing (43) are indicated by blue shading. Because of a gap in the genome sequence of the minke whale, the amino acid sequence of caspase-15 of this species could not be predicted completely. The missing amino acid residues are indicated by XXXXX. Note that several of the protein sequences have erroneously been termed “caspase-14-like” in the GenBank. Accession numbers: cattle caspase-15, AAY17561.1; orca caspase-15, XP_004277897.1; baiji caspase-15, XP_007468210.1; sperm whale caspase-15, XP_007119635.1; minke whale caspase-15, XP_007191619.1. Since the genome sequence of the dolphin appeared to be assembled incorrectly in the region of the *CASP15* gene, we amplified and sequenced parts of exon 1 (primers 5´-CCGAAGATCCCGCCGGCCATG-3´ and 5´-CAACCCTGGGAATCTCCGCGC-3´) and exon 4 (5´-TCTGGGTTTTCCTACTTGCAG-3´ and 5´-TCAGCTAAGCGTCCGCTAATG-3´) of genomic DNA from the dolphin. The resulting sequences have been submitted to the GenBank. Based on our experimental data, the following sequences correspond to exons of the *CASP15* gene of the dolphin: Exon 1, NW_004233528.1, nucl. 502-923 with corrections; exon 2, NW_004238427.1, nucl. 1431-1483; exon 3, NW_004218277.1, nucl. 15221-15072; exon 4, NW_004218277.1: 16020-15827 and nucl. 11158-11127 with corrections; exon 5, NW_004218277.1, nucl. 11535-11519; exon 6, NW_004212924.1, nucl. 40421-40312; exon 7, NW_004212924.1, nucl. 36859-36861.

```
                A  F  Q  E  E  L  A  Q  F  R  E  Q  L  D  T  C  R  G  P  V  S  C  A  L  V  A
      Human  AGGCTTTCCAGGAGGAGCTGGCCCAGTTCCGGGAGCAACTGGACACCTGCAGGGGCCCTGTGAGCTGTGCCCTTGTGGCC
     Cattle  AGACTTTCCAGGAGGAGATGGCCCAGTTCCGGAAGCGGCTGGACGCCCACAGGGGCCCCGTGAGCTGTGCCCTTGTGGCC
    Dolphin  AGACTTTCCAGGAGGAGATGGTCCATTTCTGGGAGTGGCTGGATGCCTGCAGGGACCCTGTGAGCTGTGCCCTTGTGGCC
       Orca  AGACTTTCCAGGAGGAGATGGTCCATTTCTGGGAGTGGCTGGATGCCTGCAGGGGCCCTGTGAGCTGTGCCCTTGTGGCC
Sperm whale  AGGCTTTCCAGGAGGAGATGGCCCATTTCTGGGAGTGGTTGGATGCCTGCAGGGGCCCTGTGAGCTGTGCCCTTGTGGCC
Minke whale  AGGCTTTCCAGGAGGAGATGGCCCATTTCTGGGAGTGGCTGGATGCCTGCAGGGGCCCTGTGAGCTGTGCCCTTGTGGCC

              L  M  A  H  G  G  P  R  G  Q  L  L  G  A  D  G  Q  E  V  Q  P  E  A  L  M  Q  E
      Human  CTGATGGCCCATGGGGGACCACGGGGTCAGCTGCTGGGGGCTGACGGGCAAGAGGTGCAGCCCGAGGCACTCATGCAGGA
     Cattle  CTCATGGCCCACGGGGGGCCTCAGGGGCAGCTCCTGGGGGCTGACGGGCAAGAGAGGCAGCTGGAGGTGCTTGTGCAGGA
    Dolphin  CTGATGGCCCACGGGGGGCCTCAGAGGCAGCTACTGGGTGCTGATAGGTAAGAGGTGCAGCCAGAGGCGCTGGTGCAGGA
       Orca  CTGATGGCCCACGGGGGGCCTCAGGGGCAGCTACTGGGTGCTGATAGGTAAGAGGTGCAGCCAGAGGCGCTGGTGCAGGA
Sperm whale  CTGATGGCCCATGGGGGGCCTCAGGGGCAGCTACTGGGGGCTGATAGGTGAGAGGTGCAGCCAGAGGTGCTGGTGCAGGA
Minke whale  TTGATGGCCCACGGGGGGCTTCAGGGGCAGCTACTGGGGGCTGATAGGTAAGAGGTGCAGCCAGAGGCGCTGGTGCAGGA

               L  S  R  C  Q  V  L  Q  G  R  P  K  I  F  L  L  Q  A  C  R  G
      Human  GCTGAGCCGCTGCCAGGTGCTGCAGGGCCGCCCCAAGATCTTCCTGTTGCAGGCCTGCCGTGGGGGT
     Cattle  GCTGAGCCACTGCGGGGCGCTGCGGGGCCGCCCCAAGATCTTCCTGCTTCAGGCTTGCCGTGGGGGT
    Dolphin  GCTGAGCTGCTGTAGGGCCCTGTGGGGCTGCCCCAAGATCTTCCTGCTTCAGGCTTGCCATGGG-GT
       Orca  GCTGAGCTGCTGTAGGGCCCTGTGGGGCTGCCCCAAGATCTTCCTGCTTCAGGCTTGCTGTGGG-GT
Sperm whale  GCTGAGCTGCCGTAGGGCCCTGTGGGGCCGCCCCAAGATCTTCCTGCTTCAGGCTTGCCGTGGG-GT
Minke whale  GCTGAGCTGCTGTAGGGCCCTGTGGGGCCGCCCCAAGATCTTCCTGCTTCAGGTTTGCCGTGGG-GT
```

Suppl. Fig. S9. Nucleotide sequence alignment of exon 6 of the *CASP16* gene of various species. Nucleotides identical in all species are shown in red. Mutations leading to in-frame stop codons or frame shifts are highlighted by black shading and white font. The amino acid sequence encoded by exon 6 of human *CASP16* is shown above the nucleotide sequences. The cysteine residue homologous to the active center of other caspases is highlighted by yellow shading. Note that the genomic region corresponding to this exon is absent from the *CASP16* pseudogene of the baiji (not shown). Splice acceptor (AG) and donor (GT) sites at the ends of the flanking introns are underlined.

A

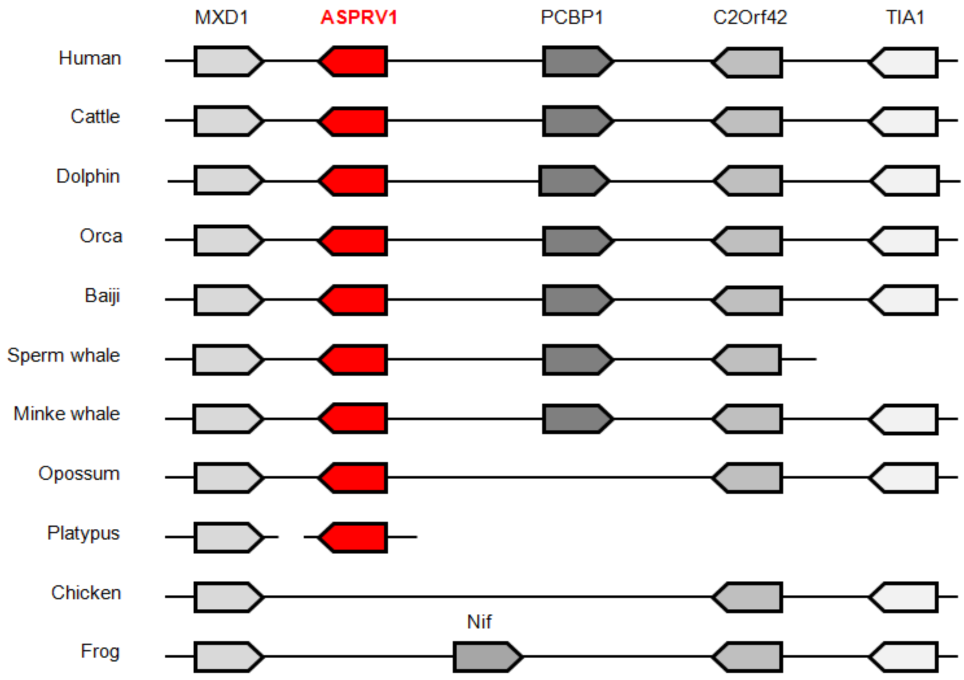

B

```
    Dolphin  MAGSAARSWEGHREHAFVLEPFDGANVAPHLWLNRFEVISDLSHWDHATKLRFLKESLRGDALEA
       Orca  MAGSAARSWEGHREHAFVLEPFDGANVAPHLWLNRFEVISDLSHWDHATKLRFLKESLRGDALEA
      Baiji  MAGSAARSWEGRREDAFVLEPFDGANVAPHLWLNXFEVISDLSHWDHATKLRFLRESLRGDALEA
Sperm whale  MAGSRARSQEGRRQYAFVLEPFDGANAAPHLWLHRFEVISDLNHWDHATKLRFLKESLRGDALEV
Minke whale  MAGSGARSREGRREHAFVLEPFDGANVAPHLWLYRFEVISDLNHWDHATKLRFLKESLRGDALEV
     Cattle  MAGSGVRSQE----RAFVPEPFDGASVAPHLWLHRFEVINHLNHWDHITKLRFLKESLRGDALGA
      Human  MAGSGARSEEGRRQHAFVPEPFDGANVVPNLWLHSFEVINDLNHWDHITKLRFLKESLRGEALGV

    Dolphin  YSGLSPEDQGEYGAVKEALLKTTGGPGAAHSYQPKEILFANSMGKGYYLKGNIGDVPVSFLVDSG
       Orca  YSGLSPEDQGEYGAVKEALLKTTGGPGAAHSYQPKEILFANSMGKGYYLKGKIGDVPVSFLVDSG
      Baiji  YSGLSPEDQGEYGAVKEALLKTSGGPGAAHGYQPKEILFTNSMGKGYYLKGKIGDVPVSFLVDSG
Sperm whale  YSGLSPEDQGEYGAVKETLLKTSGGPGVAPSYQPKEILFANSMGKGYYLKGKIGEVPVSFLVDSG
Minke whale  YSGLSPEDQGEYRAVKETLLKTFGGPGVAHSYQPKEILFANSMGKGYYLKGKIGEVPVSFLVDSG
     Cattle  FNGLSPEDQGNYEAVKETLLKTFGGPEAAHSHLPKEIVFANSMGKGYYLKGKIGKVPVRFLVDSG
      Human  YNRLSPQDQGDYGTVKEALLKAFGVPGAAPSHLPKEIVFANSMGKGYYLKGKIGKVPVRFLVDSG

    Dolphin  AQVSVVHPRLWKEVTGGDLDTLRPFENVVKVANGAEMKILGVWDTVISLGTLKLKAAFLVANASA
       Orca  AQVSVVHPRLWKEVTSGDLDSLQPFENVVKVANGAEMKILGVWDTVISLGKLKLKAAFLVANASA
      Baiji  AQVSVVHPRLWKEVTDGNLDALRPFENVVKVANGAEMKILGVWDTVISLGKLELKAAFLVADASA
Sperm whale  AQVSVVHPRLWEEVTNGDLDALRPFEDVVKVANGAEMKILGVWDTVVSLGKMKLKAAFLVAYASM
Minke whale  AQVSVVHPSLWEEVTDGDLDTLRPFENVVKVANGAELKILGVWDTEVSLGKLKLKAAFLVANASA
     Cattle  AQVSVVHPSLWEEVTDGELDTLRPFENVVKVANGAEMKILGVWDTVVSLGKLKLKAAFLVANASA
      Human  AQVSVVHPNLWEEVTDGDLDTLQPFENVVKVANGAEMKILGVWDTAVSLGKLKLKAQFLVANASA

    Dolphin  EDAIIGTDLLQDHNAVLDFEHRTCTLQGKKFRLLPVGGSLEDEFDLEFIEEELYSEEGRQQLPY
       Orca  EDAIIGTDLLQDHNAVLDFEHRTCTLQGKKFRLLPVGGSLEDEFDLEFIEEELYSEEGRQQLPY
      Baiji  EEAIIGTDLLQDQGAVLDFEHRTCTLKGMKFRLLPVGGSLEDEFDLEFIEEELYSEGGRQQLSY
Sperm whale  EEAIIGTDLLQDHNAVLDFEHRTCTLRGKKFRLLPVGGSREDEFNLDLIEEELYSEEGRQQLFY
Minke whale  EDAIIGTDMLQDHDAVLDFEHHTCTLKGKKFRLLPVGGSLEDEFDLELIEEEPYSEEGRQQLSY
     Cattle  EEAIIGTDVLQDHNAVLDFEHRTCTLKGRKFRLLPVGGSLEDEFDLELIEEEPSSEEGGQQLSC
      Human  EEAIIGTDVLQDHNAILDFEHRTCTLKGKKFRLLPVGGSLEDEFDLELIEEDPSSEEGRQELSH
```

Suppl. Fig. S10. Conservation of *ASPRV1* (saspase) in cetaceans. (**A**) Comparison of the aspartic peptidase retroviral-like 1 (*ASPRV1*) gene locus in vertebrates. Protein-coding genes at genomic loci flanked by *MXD1* and *TIA1* are shown for cetaceans, other mammals and 2 non-mammalian species. In the genome sequences of the sperm whale and the platypus, the current sequence assembly of this region is incomplete. "Dolphin" refers to *Tursiops truncatus*, and "frog" refers to *Xenopus tropicalis.* MXD1, MAX dimerization protein 1; ASPRV1, saspase; PCBP1, poly(rC) binding protein 1; C2Orf42,

chromosome 2 open reading frame 42; TIA1, TIA1 cytotoxic granule-associated RNA binding protein; Nif, low molecular weight neuronal intermediate filament. (**B**) Amino acid sequence alignment of saspase (ASPRV1) proteins. The open reading frames of ASPRV1 genes were conceptually translated, and the sequences corresponding to the 28 kDa proform of saspase (44) were aligned. Amino acid residues, that are conserved in all species, are marked by red letters. The aspartic acid residue in the catalytic center is highlighted by yellow shading. Residues corresponding to the proenzyme processing sites, as determined by Matsui et al. (45), are indicated by blue shading. The position of an unknown amino acid residue, that corresponds to an undefined nucleotide in the nucleotide sequence in the *ASPRV1* gene of the baiji, is indicated by X.

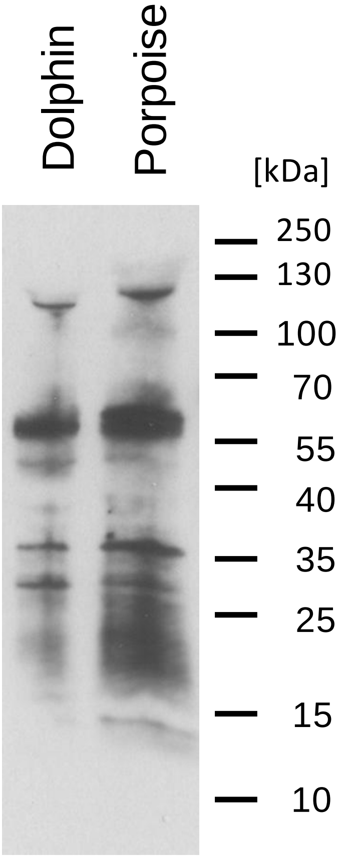

Suppl. Fig. S11. Immunoblot analysis of epidermal protein extracts using an anti-filaggrin antibody. Proteins were extracted from the epidermis of a stranded dolphin (*Tursiops truncatus*) and a stranded harbor porpoise (*Phocoena phocoena*) (tissue samples in ethanol, kindly provided by Rob Deaville, Zoological Society of London, London, UK). Immunoblot analysis was performed according to a published protocol with modifications (Fischer H, Rossiter H, Ghannadan M, *et al. Differentiation* 2005: **73**: 406-413). Tissue samples were lysed in a buffer containing 62.5 mM Tris/HCl (pH 6.8), 6 M urea, and 2% SDS, and boiled in the presence of 5% β-mercaptoethanol. The lysates were electrophoresed through an 8–18% gradient polyacrylamide gel containing SDS and blotted onto a nitrocellulose membrane. Blots were incubated with a rabbit polyclonal anti-filaggrin antibody (1:500) (Covance, Berkely, CA; catalog number: PRB-417P), followed by incubation with goat anti-rabbit-HRP (Bio-Rad, USA) and development with the enhanced chemiluminescence (ECL) detection system. The predicted molecular weight of dolphin profilaggrin is 108.3 kDa. The identity of the bands remains to be determined as the epitope of the antibody is only partly conserved in dolphins, and unspecific binding of the antibody to other proteins is possible. Of note, filaggrin fragments in the size range of 17-30 kDa have been reported for epidermal lysates of caspase-14 knockout mice (21, 56). The positions of molecular weight marker bands are indicated on the right. kDa, kilo-Dalton.

Suppl. Table S1. S100 fused-type protein (SFTP) genes of cetaceans

Species	Gene	Accession nr. of genomic sequence	Exon 1 end	Exon 2 start	CDS start	Exon 2 end	Exon 3 start	CDS end	Protein encoded	Note
Dolphin	CRNN	n.a.	n.d.	n.d.	n.d.	n.d.	n.d.	n.d.	no	
	FLG2	NW_004201070.1	n.d.	n.d.	14202	14339	15363	>15790	no	frameshift
	**FLG**	NW_004211506.1	21638	34353	34374	34511	35579	38407	**yes**	
	HRNR	NW_004211506.1	n.d.	n.d.	67642	67779	70056	73338	no	premature stop codons and frameshift in exon 3
	RPTN	NW_004203980.1	n.d.	n.d.	n.d.	n.d.	>55359	<54832	no	premature stop codons
	TCHH	NW_004220296.1	n.d.	n.d.	n.d.	n.d.	<26622	>27194	no	
	TCHHL1	NW_004199860.1	n.d.	n.d.	n.d.	n.d.	>214945	<214724	no	premature stop codon
	TCHHL2	n.a.	n.d.	n.d.	n.d.	n.d.	n.d.	n.d.	no	
Orca	CRNN	n.a.	n.d.	n.d.	n.d.	n.d.	n.d.	n.d.	no	
	FLG2	NW_004438651	n.d.	n.d.	2264261	2264124	2263105	<2261150	no	premature stop codons in exon 3
	**FLG**	NW_004438651	2229026	2216289	2216268	2216131	2215312	2212566	**yes**	sequence gap in the middle of exon 3
	HRNR	NW_004438651	n.d.	n.d.	n.d.	n.d.	2184107	<2183688	no	premature stop codon
	RPTN	n.a.	n.d.	n.d.	n.d.	n.d.	n.d.	n.d.	no	
	TCHH	NW_004438651	n.d.	n.d.	n.d.	n.d.	>2147857	<2147309	no	
	TCHHL1	n.a.	n.d.	n.d.	n.d.	n.d.	n.d.	n.d.	no	
	TCHHL2	n.a.	n.d.	n.d.	n.d.	n.d.	n.d.	n.d.	no	
Baiji	CRNN	NW_006787556.1	n.d.	n.d.	n.d.	n.d.	1312295	>1311978	no	premature stop codon
	FLG2	NW_006787556.1	n.d.	n.d.	n.d.	n.d.	1279072	>1277439	no	frameshift; premature stop codon
	**FLG**	NW_006787556.1	1245034	1219352	1219331	1219194	1218369	1215844	**yes**	
	HRNR	n.a.	n.d.	n.d.	n.d.	n.d.	n.d.	n.d.	no	
	RPTN	n.a.	n.d.	n.d.	n.d.	n.d.	n.d.	n.d.	no	
	TCHH	n.a.	n.d.	n.d.	n.d.	n.d.	n.d.	n.d.	no	
	TCHHL1	n.a.	n.d.	n.d.	n.d.	n.d.	n.d.	n.d.	no	
	TCHHL2	n.a.	n.d.	n.d.	n.d.	n.d.	n.d.	n.d.	no	
Sperm whale	CRNN	NW_006718175.1	n.d.	n.d.	n.d.	n.d.	68985	>70620	no	frameshift; premature stop codon
	FLG2	n.a.	n.d.	n.d.	n.d.	n.d.	n.d.	n.d.	no	
	FLG	n.a.	n.d.	n.d.	n.d.	n.d.	n.d.	n.d.	no	
	HRNR	n.a.	n.d.	n.d.	n.d.	n.d.	n.d.	n.d.	no	
	RPTN	NW_006717962.1	n.d.	n.d.	99953	99813	n.d.	n.d.	no	
	RPTN	NW_006717628.1	n.d.	n.d.	n.d.	n.d.	<38056	>38532	no	premature stop codons
	TCHH	NW_006716874.1	n.d.	n.d.	115803	115958	n.d.	n.d.	no	premature stop codon
	TCHHL1	NW_006712966.1	n.d.	n.d.	>463584	<463522	n.d.	n.d.	no	
	TCHHL2	n.a.	n.d.	n.d.	n.d.	n.d.	n.d.	n.d.	no	
Minke whale	CRNN	NW_006726465.1	n.d.	n.d.	2404904	2404761	2403542	<2402073	no	premature stop codons in exons 2 and 3
	FLG2	n.a.	n.d.	n.d.	n.d.	n.d.	n.d.	n.d.	no	
	FLG	n.a.	n.d.	n.d.	n.d.	n.d.	n.d.	n.d.	no	
	HRNR	NW_006726465.1	n.d.	n.d.	2355919	2355782	2353959	<2352958	no	premature stop codon in exon 3
	RPTN	n.a.	n.d.	n.d.	n.d.	n.d.	n.d.	n.d.	no	
	TCHH	n.a.	n.d.	n.d.	n.d.	n.d.	n.d.	n.d.	no	
	TCHHL1	n.a.	n.d.	n.d.	n.d.	n.d.	n.d.	n.d.	no	
	TCHHL2	NW_006726465.1	n.d.	n.d.	2286435	2286295	2283077	<2282193	no	premature stop codons in exon 3

Notes: CRNN, cornulin; FLG-2, filaggrin-2; FLG, filaggrin; HRNR, hornerin; RPTN, repetin; TCHH, trichohyalin; TCHHL1, trichohyalin-like 1; TCHHL2, trichohyalin-like 2; nr., number; n.d., not determed; CDS, coding sequnce; ORF, open reading frame. Some genomic sequence scaffolds contain genes in negative orientation, that is the 5´-end of the gene has a higher nucleotide number (within the reference genomic sequence) than the 3´-end.

Suppl. Table S2. Interspecies amino acid sequence identity (%) of filaggrin repeats and linkers

	Human	Cattle	Baiji	Orca	Dolphin
Human	100.0	41.5	35.7	35.2	35.2
Cattle	41.5	100.0	70.5	72.0	72.0
Baiji	35.7	70.5	100.0	90.5	90.5
Orca	35.2	72.0	90.5	100.0	98.5
Dolphin	35.2	72.0	90.5	98.5	100.0

Note: Amino acid sequences corresponding to the following regions of profilaggrin from different species were aligned and the percentage of positions with pairwise sequence identity were calculated: human profilaggrin (NP_002007.1) residues 1669-1869, cattle profilaggrin (XP_001255583.5) residues 1669-1869, baiji profilaggrin residues 317-515, orca profilaggrin residues 308-506 and dolphin profilaggrin residues 308-506. The aligned sequences correspond in length to 1 repeat + linker of cattle and cetacean filaggrin and to a part of the human filaggrin repeat + linker. The human filaggrin repeats are longer than those cetartiodactyls. The segment of the human filaggrin repeat which does not have a counterpart in cetartiodactyls was not included in the determination of identity values. Species: human (*Homo sapiens*), cattle (*Bos taurus*), baiji (*Lipotes vexillifer*), orca (*Orcinus orca*) and dolphin (*Tursiops truncatus*).
